# Repression of *TERMINAL FLOWER1* primarily mediates floral induction in pear (*Pyrus pyrifolia* Nakai) concomitant with change in gene expression of plant hormone-related genes and transcription factors

**DOI:** 10.1093/jxb/erx296

**Published:** 2017-09-07

**Authors:** Songling Bai, Pham Anh Tuan, Takanori Saito, Akiko Ito, Benjamin Ewa Ubi, Yusuke Ban, Takaya Moriguchi

**Affiliations:** 1 Institute of Fruit Tree and Tea Science, NARO, Tsukuba, Ibaraki, Japan; 2 Zhejiang Provincial Key Laboratory of Horticultural Plant Integrative Biology, Zhejiang University, Hangzhou, China; 3 Department of Plant Science, University of Manitoba, Winnipeg, Manitoba, Canada; 4 Graduate School of Horticulture, Chiba University, Matsudo-shi, Chiba, Japan; 5 Department of Biotechnology, Ebonyi State University, Abakaliki, Nigeria; 6 Western Region Agricultural Research Center, NARO, Division of Lowland Crop Research, Fukuyama-shi, Hiroshima, Japan; 7 Institute of Fruit Tree and Tea Science, NARO, Division of Citrus Research, Okitsu-Nakacho Shimizu, Shizuoka, Japan

**Keywords:** Floral induction, pear, plant hormone-related genes, signal transduction, *TERMINAL FLOWER1*, transcription factor

## Abstract

Floral induction is an important event in the annual growth cycle of perennial fruit trees. For pear, this event directly affects fruit production in the following year. The flower buds in many species are induced by *FLOWERING LOCUS T* (*FT*), whose effect is repressed by the meristem-expressed gene *TERMINAL FLOWER1* (*TFL1*). In this study, we investigated the functions of pear *FT* and *TFL1* genes during floral development. Expression of pear *FTs* (*PpFT1a* and *PpFT2a*) in reproductive meristems was not obviously induced prior to floral initiation, while expression of *TFL1s* (*PpTFL1-1a* and *PpTFL1-2a*) rapidly decreased. The induction of the productive meristem identity MADS-box gene *AP1* after repression of *PpTFL1s* suggested a primary role for *PpTFL1* in floral induction. RNA-seq analysis suggested that plant hormone-related genes and several transcription factors that were coexpressed with *PpTFL1* were potentially involved in the *PpTFL1*-mediated floral induction. Our data indicate the essential function of *TFL1* in pear floral induction and add another species in the family Rosaceae in addition to strawberry and rose that shows a role for *TFL1* in floral induction.

## Teaser

Repression of *TERMINAL FLOWER1* primarily mediates floral induction in pear, and plant hormone-related genes and several transcription factors contribute to this.

## Introduction

Japanese pear (*Pyrus pyrifolia* Nakai), which belongs to the tribe Pyreae of the family Rosaceae, is an important fruit in Japan with the third highest fruit production following citrus (including Satsuma mandarin and other citrus fruits) and apple. The life cycles of perennial woody species such as pear are different from those of annual plants. With regards to the growth physiology of pear trees, the plants reach reproductive maturity at the end of their juvenile phase, which requires about 8–12 years (Layne and Quamme, 1975). Once reproductive maturity is obtained, flower buds are formed every year. From June to August, visible floral initiation for the next growth season takes place concomitantly with the arrest of shoot elongation (Ito *et al.*, 1999). In autumn, the leaves fall and an endodormancy stage starts during which buds cannot sprout even under suitable growth conditions until a chilling requirement is fulfilled (Lang *et al.*, 1987). After fulfilment of the chilling requirement, the dormancy status changes to ecodormancy during which buds can potentially start to sprout, but environmental conditions in January–February still hinder sprouting (Faust *et al.*, 1997). When the temperature becomes warmer in the spring, the buds sprout and bloom and the development of new leaves and shoot elongation start (Saito *et al.*, 2015) together with fruit development. Thus, for perennially woody plants like pear, vegetative and reproductive growth proceed simultaneously in the same year. In this way, the annual cycle of pear growth is highly regulated and coordinated by internal and external signals.

During the annual growth cycle, floral development is an important step for fruit production. Pear flower buds contain both leaf and flower primordia, known as mixed buds (we use the term ‘flower bud’ for convenience). Floral development has three steps. First, the vegetative meristem shifts to reproductive phase in response to floral-inductive cues (floral induction), and then transforms into a visible dome-like structure (floral initiation). After floral initiation, the meristem subsequently develops into floral organs (floral differentiation). Visible floral initiation of pear starts in June to August in Japan, depending on the shoot type; it occurs earlier in flower buds of spurs than in those of long shoots. The apical buds in the spur form flower buds in the middle of June but the initiation of flower buds in the newly grown long shoots is more complex. Usually the apical buds and several distally positioned lateral buds can form the flower buds, while others develop into leaf buds. The number of the flower buds varies among shoots, depending largely on their nutritional conditions.

Regarding external signals for floral induction, Tromp (1980) demonstrated that a higher ambient temperature (24 °C) delays flower bud production in apple. As apple is genetically close to pear, this finding suggests that climate change and extreme weather in summer might hamper this step in pear, resulting in a reduced number of flowers in the following spring (Tromp, 1976; unpublished data). By contrast, far-red light treatment, which may be comparable to short-day treatment (Olsen and Junttila, 2002), has been shown to enhance flower bud production in potted pear trees (Ito *et al.*, 2014). From these results, one might suppose that either or both temperature and day length are involved in floral induction in pear. Even so, it is not clear which range of temperatures and/or day lengths is critical for this step. Alternatively, the possibility cannot be ruled out that floral induction is not regulated by external cues but controlled by internal rhythms like the circadian clock. Thus, the molecular mechanism underlying floral induction in pear has not yet been fully elucidated. Therefore, molecular insights would be helpful for commercial fruit production of pear, for example, for increasing the numbers of flower buds even under warmer global temperatures.

It has been well known that several factors, among which photoperiod is the most important, regulate the flowering time of other plants (Andrés and Coupland, 2012). The genes that regulate the transition from the vegetative phase or alter meristem identity have been isolated and characterized in model plants (Bradley *et al.*, 1997; Kobayashi *et al.*, 1999; Parcy *et al.*, 2002). In particular, *FLOWERING LOCUS T* (*FT*) is a key regulatory gene of floral transition in Arabidopsis and other herbaceous species (Kobayashi *et al.*, 1999; Danilevskaya *et al.*, 2008). *FT* mRNA is transcribed and translated in leaves, and FT protein is then translocated through the phloem to the shoot apex (Corbesier *et al.*, 2007; Tamaki *et al.*, 2007). FT triggers floral development by interacting with the bZIP protein FD (Abe *et al.*, 2005) via a 14-3-3 protein (Taoka *et al.*, 2011) to activate the downstream transcriptional factor *SUPPRESSOR OF OVEREXPRESSION OF CONSTANS 1* (*SOC1*) and the MADS-box genes, including *APETALA1* (*AP1*), *FRUITFUL* (*FUL*), and *CAULIFLOWER* (*CAL*) (Ferrandiz *et al.*, 2000), responsible for inflorescence meristem identity. Conversely, *TERMINAL FLOWER1* (*TFL1*) is involved in maintaining vegetative meristem identity by preventing the expression of *AP1* and *LEAFY (LFY*) (Ratcliffe *et al.*, 1999). In some woody plants, the *FT* gene was duplicated to coordinate with the seasonal flowering cycle (Hsu *et al.*, 2011). In poplar, *PtFT2* supports vegetative growth and inhibits bud set during autumn, while *PtFT1* initiates reproductive growth in summer (Hsu *et al.*, 2011). Similarly, the two apple *FT* homologs have diverse roles in reproductive growth induction. *MdFT1* is expressed mainly in apical buds and functions in the induction of flower buds, while *MdFT2* is mainly expressed in reproductive organs and has functions in the regulation of differentiation of flower buds and induction of young fruits, although both apple MdFTs can induce flowering in transgenic Arabidopsis (Kotoda *et al.*, 2010). We have cloned two pear *FT* genes from Japanese pear (Ito *et al.*, 2014, 2016), but their functions during floral induction have not been well characterized. In addition, plant hormones, such as auxin, cytokinin (CK), abscisic acid (ABA), gibberellic acid (GA), and ethylene, also play important roles in the regulation of bud growth and floral induction in woody plants (Han *et al.*, 2007; Wilkie *et al.*, 2008; Mutasa-Göttgens and Hedden, 2009; Lee and Lee, 2010) although the detailed pathway is still obscure. A recent study showed that auxin, CK, GA, and ABA levels significantly changed during the shoot-bending-induced flower bud initiation along with the alteration of the expression of flowering control genes in apple (Xing *et al.*, 2016).

In this study, we show that pear *FT* (*PpFT1a* and *PpFT2a*) expression was not obviously induced during floral induction in apical meristems, while *TFL1* (*PpTFL1-1a* and *PpTFL1-2a*) expression decreased prior to the visible initiation stage, which was followed by induction of the meristem identity-related MADS-box gene *AP1* (*PpAP1*), suggesting a primary role for *PpTFL1* is in floral induction. Furthermore, several hormone-related genes and transcription factors identified via RNA-seq could potentially contribute to *PpTFL1*-mediated floral induction at the transcriptional level. This work confirms that pear represents another example within the family Rosaceae, in addition to strawberry and rose, of TFL1 functioning as a major regulator of floral induction, and encourages us to further study the underlying mechanism of floral development in pear.

## Materials and methods

### Plant material

The buds were collected from Japanese pear (‘Kosui’) grown at the Institute of Fruit Tree and Tea Science, NARO, Tsukuba, Japan (36 °N, 140 °E). All samples were collected between 08.00 and 09.00 h. For gene expression analysis, apical tips/buds of the newly grown long shoots (shown in Supplementary Fig. S1A at *JXB* online) whose buds always develop into flower buds under natural conditions were collected at each of the five flower developmental stages in 2011 (21 June, 5 July, 19 July, 2 August, and 16 August), 2012 (27 June, 9 July, 19 July, 30 July, and 9 August), and 2014 (20 June, 8 July, 18 July, 4 August, and 13 August). In addition, apical tips/buds of the spurs (length <3 cm; Supplementary Fig. S1B) were collected at six time points (20 May, 29 May, 9 June, 19 June, 29 June, and 9 July) in 2009. Proximal lateral leaf buds of the newly grown long shoots (low positioned buds), which never develop into flower buds, were collected in 2009. The collected apical tips/buds were immediately frozen in liquid nitrogen and stored at −80 °C until they were used for RNA extraction.

### Phylogenetic tree analysis

Sequences of *FT*-like and *TFL1*-like genes deposited in the National Center for Biotechnology Information (NCBI) database were retrieved for the assembly of a phylogenetic tree. The sequences were aligned using the ClustalW algorithm and manually edited in Geneious (Biomatters Ltd, Auckland, New Zealand). Tree building with the maximum likelihood method was performed using the PHYML plugin and the tree builder provided in Geneious. To test the robustness of the tree, 1000 bootstrap replicates were carried out.

### Quantitative reverse-transcription PCR (qRT-PCR)

Total RNA was isolated from the ‘Kosui’ tissues collected during flower development, as previously described (Wan and Wilkins, 1994). At least 30 buds were collected and used for RNA isolation for each sample. First-strand cDNA synthesis was performed using the SuperScript^TM^ III First Strand Synthesis System (Life Technologies, Carlsbad, CA, USA). A total of 5.0 µg of RNA was first treated with DNase (Promega, Madison, WI, USA) and then reverse-transcribed using SuperScript III oligo (dT) 20 primers, according to the manufacturer’s instructions (Life Technologies). The cDNAs used for RNA-seq confirmation were synthesized from 500 ng total RNA with a SuperScript VILO cDNA Synthesis Kit (Life Technologies) following the manufacturer’s instructions. The specific primers for qRT-PCR for all genes used in this study are shown in Supplementary Table S1. As an internal control, a *PpHistoneH3a* or *PpSAND*-specific primer pair was used (Saito *et al.*, 2013; Imai *et al.*, 2014). The real-time quantification of the first-strand cDNA was performed using a 7500 Real Time PCR System (Applied Biosystems, Tokyo, Japan) and analysed with 7500 Software v. 2.0 (Applied Biosystems). For the control reactions, no template was added to the reaction mixture, which resulted in no detectable fluorescence signal. The PCR conditions were set as follows: initial denaturation for 10 s at 95 °C, followed by 40 cycles of denaturation at 95 °C for 5 s and annealing and extension for 34 s at 60 or 62 °C.

### Rescue of the late- and early-flowering Arabidopsis phenotypes, *ft10* and *tfl1-13*, by overexpression of *PpFT1a* and *PpTFL1-2a*, respectively

The Arabidopsis mutants *ft10* (Yoo *et al.*, 2005) and *tfl1-13* (ABRC No. CS6237) were cultivated in a chamber with a 16/8 h light/dark cycle at 21 °C. *PpFT1a* and *PpTFL1-2a* were amplified using primers (see Supplementary Table S1) with KOD plus NEO DNA polymerase (Toyobo, Osaka, Japan). The purified PCR product was inserted into the pENTR-D-TOPO vector (Life Technologies) according to the manufacturer’s instructions. The inserts were then recombined into the destination vector pGWB2 (Nakagawa *et al.*, 2007) using Gateway technology (Life Technologies). The resulting construct was introduced into *Agrobacterium* LBA4404 strain, and then 35S-promoted *PpFT1a* (pGWB2-PpFT1a) or *PpTFL1-2a* (pGWB2-PpTFL1-2a) cassettes were transformed into Arabidopsis mutants *ft10* or *tfl1-13* using the flower-dip method (Clough and Bent, 1998).

### Developmental stage of flower buds

The bud developmental stage was determined microscopically in five randomly selected apical buds in each batch of samples. Bud scales were removed with forceps and apical meristems were observed directly through an Olympus SZX7 microscope. The stage of bud development at each sampling date was scored on a scale of 0–10 as follows: 0: vegetative; 1: early stage of initiation (floral initiation, doming of the apex); 2: middle stage of initiation; 3: final stage of initiation; 4: lateral flower bud apparent; 5: bract and lateral flower buds beginning to develop; 6: bract and lateral flower buds forming; 7: bract and lateral flower buds well developed; 8: sepal primordia developing; 9: petal and stamen primordia forming; 10: petal and pistil forming. Supplementary Fig. S2 shows some representative photographs for stages 0, 1, and 5 of the flower buds.

### RNA-seq

We used flower buds showing developmental stage scores of 0, 0.2, and 2.0 (see above definition) from those collected in 2012 and 2014, which corresponded to 27 June, 19 July, and 30 July in 2012 and 20 June, 18 July, and 4 August in 2014, respectively. The RNA-seq analysis consisted of three stages, and samples from two different years were used as biological replicates. Total RNAs were extracted from the ground powder of at least 30 buds for each sample. Ten micrograms of total RNA each was used for next-generation sequencing. The library construction and sequencing were performed by BGI (Shenzhen, China) using the HiSeq™ 2000 (Illumina, San Diego, CA, USA) platform with a 100 bp pair-end strategy. The sequencing data have been deposited in the NCBI Sequence Read Archive (http://www.ncbi.nlm.nih.gov/sra/, accessed on 13 August) with the accession ID SRP082601.

### Sequence data processing and mapping of reads to the pear genome

To obtain high-quality clean read data for sequence analysis, certain undesirable sequences, such as adaptor sequences, empty reads, low-quality sequences with greater than 5% N (the percentage of nucleotides in the reads that could not be sequenced) and sequences containing more than 10% ambiguous bases, were removed. The clean reads were mapped to the pear genome sequence (http://peargenome.njau.edu.cn/, accessed on 13 August) using TopHat (Trapnell *et al.*, 2012) with default parameters. The reads were then assembled into transcripts and compared with reference gene models using Cufflinks (Trapnell *et al.*, 2012).

### *De novo* assembly of the transcriptome

The clean reads were assembled with Trinity (Grabherr *et al.*, 2011), and TGICL (Pertea *et al.*, 2003) was used to optimize the original Trinity assembly results by removing the sequences that could not be extended on either end. Such sequences were defined as unigenes. The unigenes were then clustered according to sequence similarity using Phrap (Gordon *et al.*, 2001). Thus, the unigenes were divided into two classes: one with clusters of several unigenes with >70% similarity among them and the other with singletons, with the prefix of Unigene. The *de novo* assembled transcripts were then merged with the genome-guided assembly. After further assembly by CAP3 (http://seq.cs.iastate.edu/cap3.html, accessed on 13 August) and removal of duplicated sequences, 74 897 transcripts remained. All of the transcripts were annotated against protein databases, including the NCBI non-redundant databases, using the BLASTX algorithm with a cutoff e-value of 10^–5^.

### Identification of significant differentially expressed genes

The clean reads were aligned to the assembled transcriptome with Bowtie (Langmead *et al.*, 2009). The reads per kilobase per million mapped reads (RPKM) value of each gene was determined by eXpress (Roberts and Pachter, 2013). Transcript abundance differences between each pair of samples were then calculated using DESeq (Anders and Huber, 2010) based on the output of eXpress. Genes with a |log_2_ratio| ≥2 and a false discovery rate significance score <0.05 were considered significantly differentially expressed. The differentially expressed genes (DEGs) were then used for co-expression network analysis using the R package WGCNA (Langfelder and Horvath, 2008). The pathway analysis of DEGs was carried out with MapMan (Usadel *et al.*, 2005).

## Results

### *PpTFL1*, but not *PpFT*, may be responsible for floral induction in pear

**Fig. 1.**
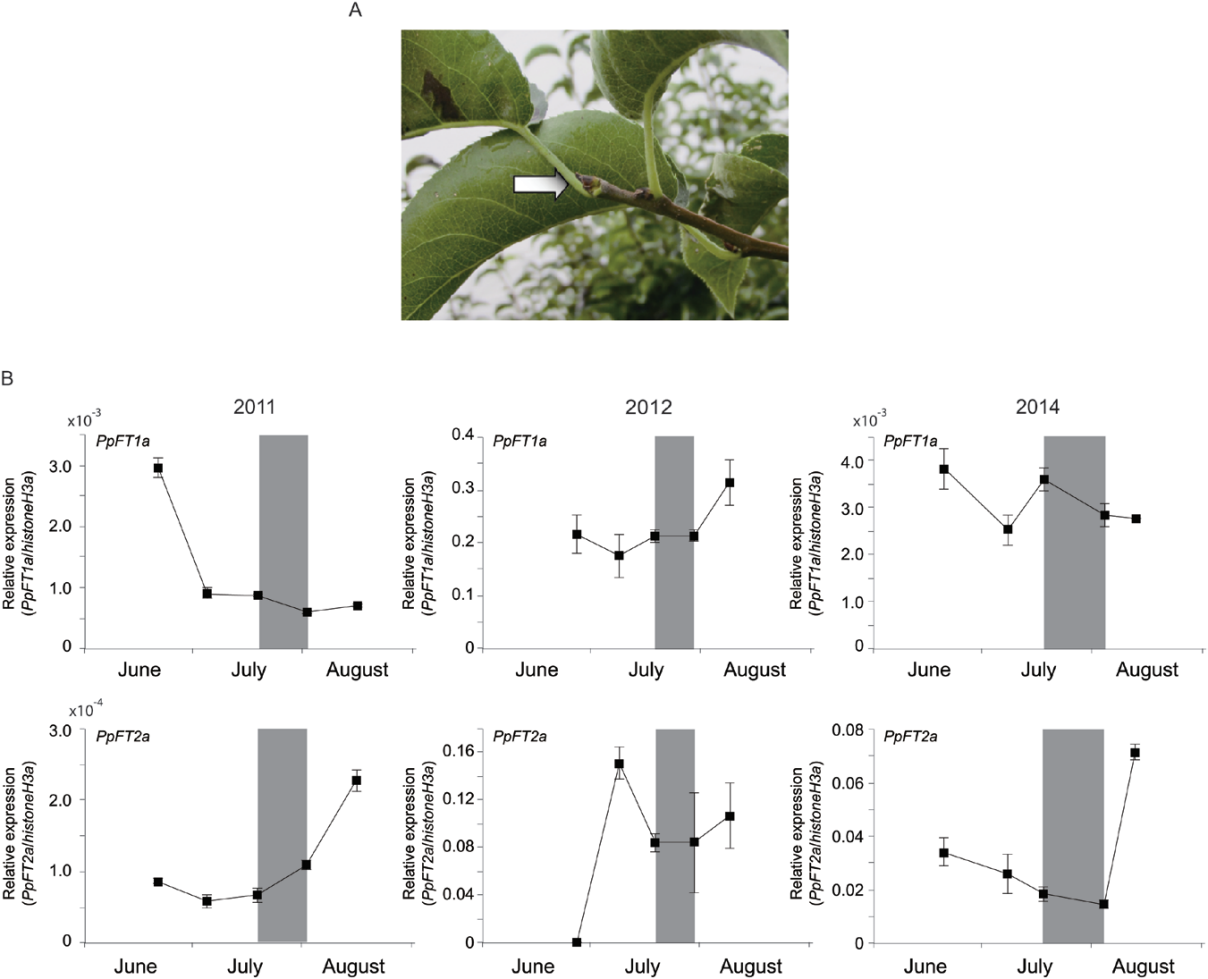
Apical tips/buds of newly grown long shoots (A; white arrow) and relative expression levels of *PpFT1a* and *PpFT2a* genes in ‘Kosui’ from June to August in 2011, 2012, and 2014 (B). Error bars indicate standard error of three technical replicates. The stage that a dome-like structure (indicating occurrence of visible floral initiation) appeared is indicated by the grey zone. (This figure is available in color at *JXB* online.)

In phylogenetic tree analysis, FTs from woody Rosaceae species were clustered together. Specifically, pear FT homologs PpFT1a and PpFT2a (Ito *et al.*, 2014, 2016) were clustered with apple MdFT1 and MdFT2, respectively (see Supplementary Fig. S3). In previous reports, *MdFT1* induction was observed from the period of flower induction to the early stage of flower development in buds but not in leaves (Kotoda *et al.*, 2010). Considering the close relationship between pear and apple, we used *PpFT1a* in this study to investigate whether overexpression of *PpFT1a* could rescue the late-flowering phenotype of the Arabidopsis *FT* null mutant, *ft10*. Successful transformation and expression of *PpFT1a* was verified in two independent lines, nos 1 and 2, by genomic PCR and qRT-PCR (Supplementary Fig. S4). Both lines showed flowering earlier than *ft10*, indicating the successful rescue of the mutation of the Arabidopsis *FT* gene. This result indicated that *PpFT1a* was a functional *FT* homolog at least in Arabidopsis. We then investigated the expression pattern of *PpFT1a* and *PpFT2a*, whose homolog in apple, *MdFT2*, can induce flowering in transgenic Arabidopsis (Kotoda *et al.*, 2010). Because the apical buds of the newly grown long shoots (shown in Fig. 1A and Supplementary Fig. S1A) always form the flower buds, we collected their apical tips/buds from June to August. Visible floral initiation, confirmed by the appearance of a dome-like structure, was recorded between mid July and early August in 2011, 2012, and 2014. qRT-PCR revealed an inconsistent expression pattern of both *PpFT1a* genes and their expression was not enhanced prior to floral initiation (Fig. 1B). The highest transcript levels of *PpFT2a* were observed in mid August, corresponding to the floral differentiation stage in 2011 and 2014 but not in 2012 (Fig. 1B). Despite this observation in those two years, our result was consistent in that neither *PpFT1a* nor *PpFT2a* was induced during floral induction in all three seasons (Fig. 1B) and in the apical buds of the spur collected in 2009 (see Supplementary Figs S1B and S5). Moreover, no obvious induction of *PpFT1a* expression was observed in the leaves or stems of the spurs (Supplementary Figs S1B and S6), suggesting that the induced *FT* expression in leaves, that was observed in Arabidopsis, may not contribute to floral induction of pear. Thus, in contrast to the high expression of *FT* in apple (Kotoda *et al.*, 2010) and Satsuma mandarin (Nishikawa *et al.*, 2007), our results showed that pear *FT* expression was not induced concurrently with floral induction, indicating that the transcriptional induction of *PpFT* might not be necessary for this step in pear.

**Fig. 2.**
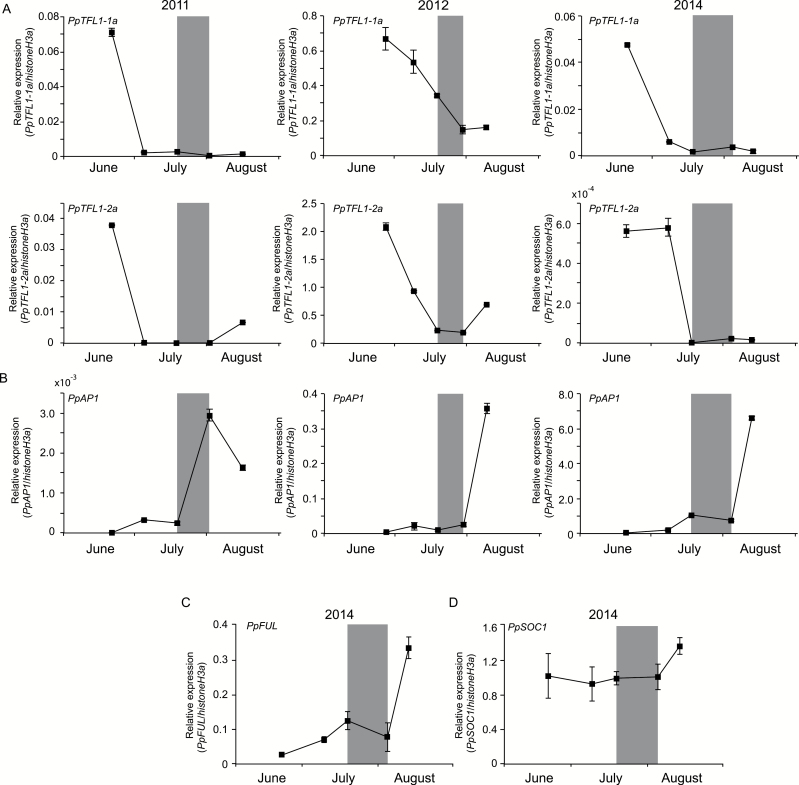
(A, B) Relative expression levels of *PpTFL1-1a*, *PpTFL1-2a* (A), and the meristem identity MADS-box genes *PpAP1* (B) in the apical tips/buds of the newly grown long shoots in ‘Kosui’ from June to August in 2011, 2012, and 2014. (C, D) The relative expression levels of *PpFUL* (C) and *PpSOC1* (D) in 2014. Error bars indicate standard error of three technical replicates. The stage that dome-like structure (indicating occurrence of visible floral initiation) appeared is indicated by the grey zone.

As Arabidopsis TFL1 functions as an inhibitor of floral induction via preventing the meristem from assuming a floral identity (Ahn *et al.*, 2006), we further characterized the functions of pear *TFL1* genes. Phylogenetic tree analysis showed that PpTFL1-1a and PpTFL1-2a were the homologs of AtTFL1 and FvKSN, which were characterized as flowering repressors, while some other genes were also clustered with AtACT (see Supplementary Fig. S3). We used *PpTFL1-2a* as the representative pear *TFL1* gene for overexpression in Arabidopsis mutant *tfl1-13*. Overexpression of *PpTFL1-2a* strongly delayed the flowering time of *tfl1-13*, making it even later than in the wild-type (Supplementary Fig. S7). The phenotypes of the overexpression lines showed disordered flower organ development similar to that in *AtTFL1* overexpression lines (Ratcliffe *et al.*, 1998), demonstrating that at least *PpTFL1-2a* was a functional homolog of the Arabidopsis *TFL1* gene. We then conducted an expression analysis of *PpTFL1-1a* and *PpTFL1-2a* along with the other floral development-related MADS-box genes, such as *PpAP1* (= *PpMADS2-1*, *AP1* homolog, GeneBank: AB623159.2), *PpFUL* (= *PpMADS3-1*, *FUL* homolog, GeneBank: AB623165.2), and *PpSOC1* (= *PpMADS5-1*, *SOC1* homolog, GeneBank:AB623161.2) (Ubi *et al.*, 2013) in the apical tips/buds of the newly grown long shoots. The expression levels of both *PpTFL1-1a* and *PpTFL1-2a* were high in June, but were rapidly reduced with the approach of the visible floral initiation stage (Fig. 2A). In contrast, the expression levels of *PpAP1* were up-regulated after floral initiation (Fig. 2B). Similar expression patterns were recorded in the apical buds of the spur from 2009 (Supplementary Fig. S5), which demonstrated the consistent expression patterns of these genes in different shoot types. Moreover, the expressions of *PpFUL* and *PpSOC1* were also elevated after floral initiation (Fig. 2C, D). These results suggested that *PpTFL1* may play a predominant role in floral induction while *PpFT* does not seem to have the primary role that it plays in other plants. We then attempted to identify genes that were possibly involved in *PpTFL1*-mediated floral induction via RNA-seq.

### RNA-seq analysis of pear buds during floral development shows distinct clusters of genes at each stage

**Fig. 3.**
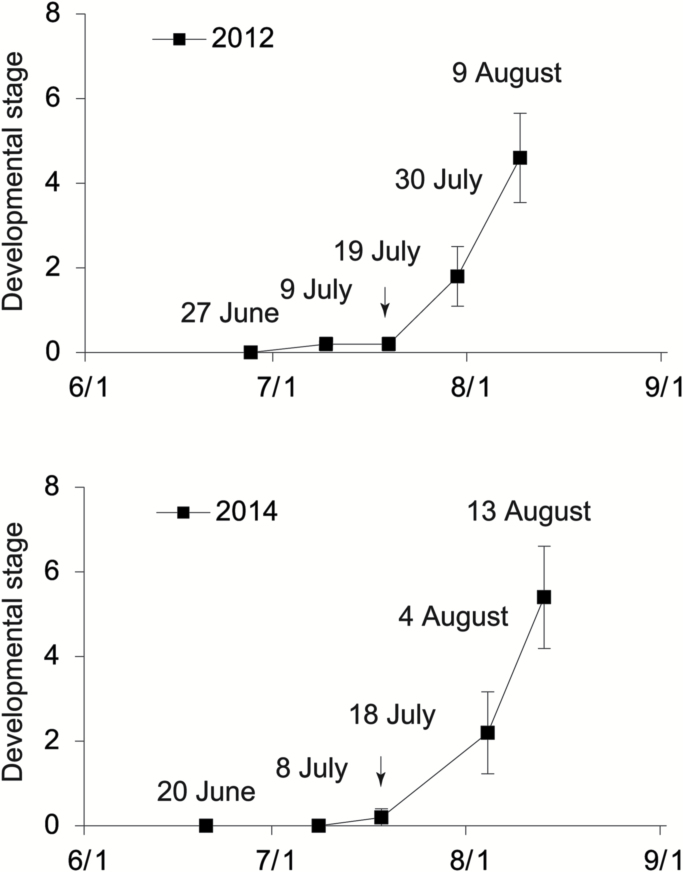
Vegetative and reproductive phase changes during floral development of apical buds from spurs of ‘Kosui’ in 2012 and 2014. Visible floral initiation of ‘Kosui’ in the apical buds of the newly grown long shoots occurred during mid to late July (arrows).

In Tsukuba, Japan, pear floral development starts in June and lasts for about one and a half months. We recorded the dynamic states of floral development in 2012 and 2014 by observing five randomly harvested apical buds of the newly grown long shoots that were destined to develop into flower buds (Fig. 3). In late June, there were no observable morphological changes in the buds (stage 0), while in mid to late July, the visible floral initiation started (stage 0.2) and then floral differentiation lasted until mid August. The transcriptomes of samples at developmental stages 0, 0.2, and 2.0 from both 2012 and 2014 were analysed; six samples in total. High-throughput sequencing generated 49.42–54.72 million (M) pair-end reads of 100 bp length from each library. After a stringent quality filtering process, 28.64 Gb of clean data (92.55% of the raw data) remained, with a Q20 percentage >98%. The counts of clean reads per library ranged from 46.29 to 49.03 M (see Supplementary Table S2). As some important genes involved in flowering, such as *PpFT1a*, were not present in the published pear genome sequence (due to the incomplete nature of the published genome), we assembled the bud transcriptomes using both the reference-guided strategy and the *de novo* strategy (refer to ‘Materials and methods’). The two resultant datasets were then merged by removing duplicated genes and genes with high similarity. Finally, 74 897 transcripts were obtained.

**Fig. 4.**
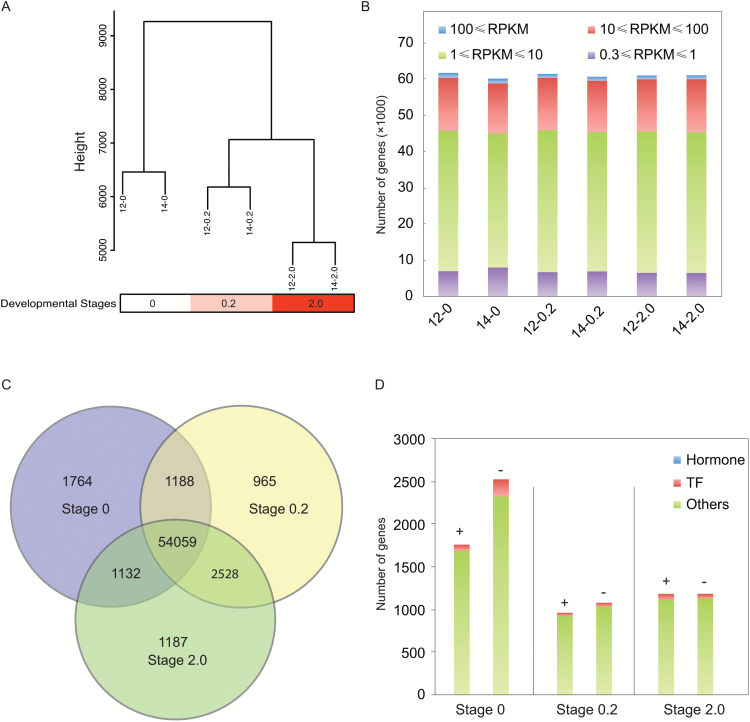
Analysis of global gene expression during floral development. (A) Cluster dendrogram showing global relationships between biological replicates and among different developmental stages. Samples were named in the format ‘year-developmental stage’; for example, ‘12–0.2’ indicates the flower bud sample of developmental stage 0.2 harvested in 2012. Note that the same stages from different years were clustered together. (B) Numbers of genes expressed in each library with RPKM>0.3. (C) Venn diagram showing the number of commonly and uniquely expressed genes among the developmental stages. (D) Developmental-stage-specifically expressed genes (+) and genes that were not expressed (−). Plant hormone-related genes and transcription factors are indicated with different shades/colors. The number of plant hormone-related genes is too low to be see in the graph. (This figure is available in color at *JXB* online.)

The RPKM of each transcript was determined by eXpress. Correlation analysis showed that the global gene expression profiles of the buds in the same stage from different years were clustered (Fig. 4A). Specifically, the buds of stage 0 showed clearly different transcriptome profiles from the buds of stage 0.2 and 2.0. In all these datasets, the RPKM value distribution showed a similar pattern among the stages with values for most genes ranging between 1 and 100 (Fig. 4B). During floral development, 72% of genes were commonly expressed in all stages, while several hundreds to thousands of genes were specifically expressed in one or two stages (Fig. 4C), including plant hormone metabolism- and signaling-related genes and transcription factors (Fig. 4D).

### Analysis of DEGs shows similar expression patterns between RNA-seq and qRT-PCR

**Fig. 5.**
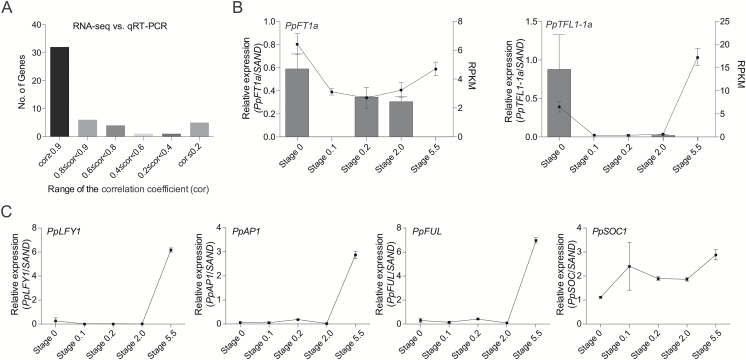
qRT-PCR confirmation of RNA-seq results for selected genes. (A) Fifty genes (Supplementary Table S3) were used for qRT-PCR confirmation using the same samples as those used for RNA-seq. The correlation coefficients (cor) between the normalized expression values of RNA-seq and qRT-PCR were calculated with the CORREL function in Excel. Note that the correlation coefficients of most genes were >0.8. (B) RPKM values (box) and relative expression identified by qRT-PCR (bar) for *PpFT1a* and *PpTFL1-1a*. (C) Relative expression of the floral differentiation-related genes *PpLFY1 PpAP-1*, *PpFUL*, and *PpSOC1*. Data are presented as averages of samples of the same developmental stage from 2012 and 2014. Error bars indicate standard error using the two years’ samples.

DEGs were identified in each pair of samples with DESeq using the criteria mentioned in ‘Materials and methods’ and filtered in advance using the following criterion that the highest RPKM in all the samples was lower than 0.3. At the end, 2376 DEGs remained for further analysis. Over 60 DEGs were randomly chosen for measurement of mRNA levels by qRT-PCR, among which over 70% showed a similar expression pattern between the RNA-seq and qRT-PCR data (correlation ≥0.8), indicating good reproducibility between the transcript profiles assayed by RNA-seq and the expression abundance revealed by qRT-PCR data. Here, we show the results of 50 selected genes (see Supplementary Table S3) whose expressions patterns from RNA-seq matched those from qRT-PCR (Fig. 5A).

Some important flowering-related genes were also selected for qRT-PCR analysis using the same samples as that used for RNA-seq. In accordance with our previous observation, *PpFT1a* expression was not up-regulated during floral induction; instead, it decreased from stage 0 to stage 0.2. Expression of *PpTFL1-1a* fell to almost zero in RNA-seq, which was in accordance with the qRT-PCR analysis, but increased in the later stages of floral differentiation (Fig. 5B). In addition, the expression of *PpAP1*, *PpLFY1* (Esumi *et al.*, 2007), and *PpFUL* increased after visible floral initiation (Fig. 5C), in accordance with their putative functions in flower organ development, but they were not fate determinants. Interestingly, the expression patterns of *PpSOC1*, which is also a positive regulatory candidate for flowering time determination, did not show obvious correlations to floral induction and later development (Fig. 5C).

### Identification of genes coexpressed with *PpTFL1*

**Fig. 6.**
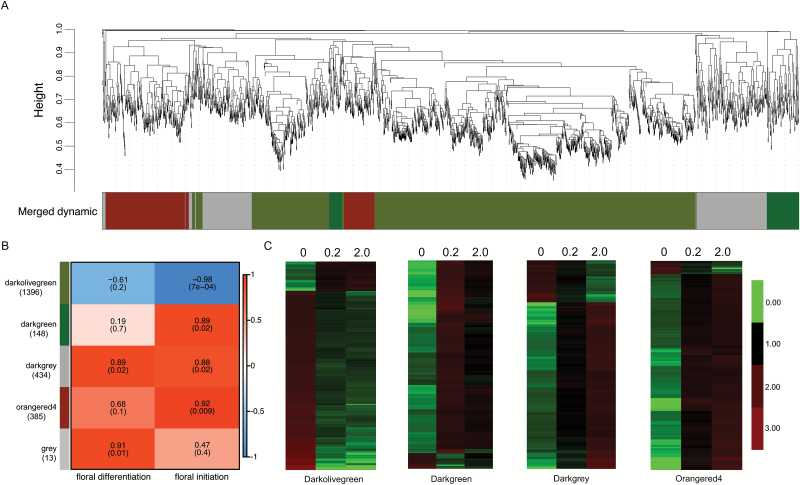
Weighted gene co-expression network analysis (WGCNA) of differentially expressed genes (DEGs) identified from ‘Kosui’ flower buds during three developmental stages. (A) Hierarchical cluster tree showing three modules of coexpressed genes. Each of the 2376 DEGs represents a leaf in the tree, and each of the three modules represents a major tree branch. The lower panel shows the modules in the designated colors. (B) Module–floral initiation stage correlations and corresponding *P*-values (in parentheses). The left panel shows the three modules and the number of genes in each module. The color scale on right shows module–trait correlation from –1 to 1. In the left panel, ‘floral differentiation’ is the floral developmental stages. In the right panel, ‘floral initiation’ is the binarized developmental stages with stage 0=0 and stages 0.2 and 2.0=1. (C) Heat maps showing the expression patterns of each module. (This figure is available in color at *JXB* online.)

According to the floral development steps that were defined previously, we used two sets of trait data for further study: ‘floral differentiation’ and ‘floral initiation’. ‘Floral differentiation’ used developmental stage data (stage 0, stage 0.2, and stage 2.0) that treated the floral differentiation quantitatively, while ‘floral initiation’ qualitatively treated the flower bud developmental stages as ‘before initiation’ (developmental stage 0, assigned value 0) and ‘after initiation’ (developmental stages 0.2 and 2.0, assigned value 1). Weighted gene coexpression network analysis (WGCNA) with non-redundant DEGs identified five WGCNA modules (Fig. 6A). Module–trait relationship analysis identified ‘darkgrey’ and ‘darkolivegreen’ as most highly related to ‘floral differentiation’ and ‘floral initiation’, respectively (Fig. 6B). The expression levels of genes peaked in different floral developmental stages (Fig. 6C). As our study focused on the floral initiation process, we further analysed the genes within the module ‘darkolivegreen’ (*r*=0.92, *P*=0.009), which was the most-related module to ‘floral initiation’ (Fig. 6B). The genes belonging to the module ‘darkolivegreen’ showed higher (or lower) transcription in stage 0 and similar transcription in stages 0.2 and 2.0, indicating that these genes potentially function in floral initiation (Fig. 6C). In addition, the module ‘darkolivegreen’ was composed of 1396 genes, accounting for half of all the redundant DEGs, suggesting great internal changes during the transition to flower buds. As *TFL1* belonged to the module ‘darkolivegreen’, we considered these 1396 genes putative coexpressed genes of *PpTFL1*.

### Functional annotation of the genes coexpressed with *PpTFL1* revealed the involvement of many plant hormone-related genes

**Fig. 7.**
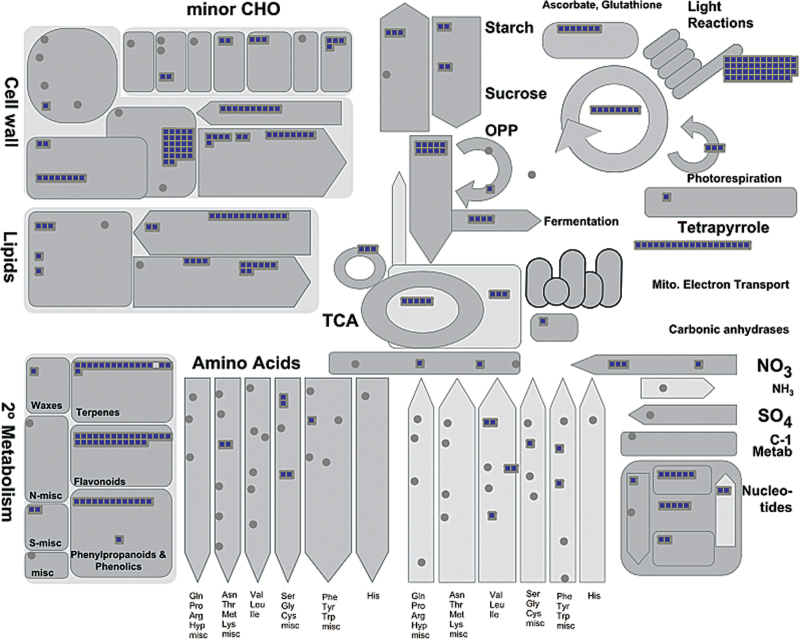
Pathway analysis of the *PpTFL1* coexpressed genes. The graph is the output of MapMan. (This figure is available in color at *JXB* online.)

Functional annotation of the *PpTFL1* co-expressed genes was carried out using MapMan. Based on this analysis, these genes were placed in some primary metabolic pathways, including photosynthesis, light reactions, lipid biosynthesis, and cell wall degradation (Fig. 7). Additionally, genes belonging to the carotenoid, flavonoid, and lignin biosynthesis pathways were also observed in this gene set (Fig. 7).

To determine the functions of plant hormones in *PpTFL1*-mediated floral induction, we focused on plant hormone metabolism- and signal transduction-related genes with synergistic (or antagonistic) expression patterns to *PpTFL1*. Genes associated with auxin, CK, ABA, and ethylene were observed during floral induction (Table 1). Notably, no genes related to GA metabolism or signal transduction were identified as DEGs. Two *9-cis-epoxycarotenoid dioxygenase* (*NCED*) genes were down-regulated to less than one-fifth expression when visible floral initiation started and *ABA 8′-hydroxylase (ABA8h'*) was also strongly down-regulated. The up-regulation of two *1-aminocyclopropane-1-carboxylate oxidase* (*ACO*) genes, which catalyse the last step of ethylene biosynthesis, was observed along with similar expression patterns for two ethylene receptors. In addition, the genes *indole-3-pyruvate monooxygenase* (*YUCCA*) and *cytokinin riboside 5′-monophosphate phosphoribohydrolase* (*LOG*) showed down-regulation like *PpTFL1*, while *adenylate isopentenyltransferase* (*IPT*) *3* was up-regulated.

**Table 1.** *Hormone-related genes that showed a coexpression pattern with* PpTFL1 Values of fold change less than 1 signify up-regulation and values more than 1 indicate down-regulation during floral induction. Genes mentioned in the main text are marked in bold. N/A, not available.

Gene id	Annotation in pear genome database in Genbank	Sequence description	Fold change (stage 0/ stage 0.2)
Auxin			
** CL11716. Contig2_All**	**XP_009360775.1**	**Probable indole-3-pyruvate monooxygenase YUCCA**	**2.593236726**
CL5495.Contig1_All	XP_009361697.1	Auxin-binding protein ABP19a	5.937830235
TCONS_00032086	XP_009353580.1	Auxin-responsive protein IAA21-like	2.079878128
TCONS_00049929	XP_009339008.1	Auxin-induced protein IAA6	11.74525523
Contig793	XP_009364491.1	Auxin-responsive protein IAA4-like	2.026782833
ABA			
** CL13198. Contig2_All**	**N/A**	**Probable 9-*cis*-epoxycarotenoid dioxygenase NCED5**	**6.862296765**
Contig4569	XP_009376987.1	Putative protein phosphatase 2C-like protein 44	2.715771741
** TCONS_00006524**	**XP_009364364.1**	**Abscisic acid 8′-hydroxylase 2**	**9.668865776**
** Unigene2348_All**	**XP_009360900.1**	**Probable 9-*cis*-epoxycarotenoid dioxygenase NCED5**	**5.707881397**
Cytokinin			
** CL12024. Contig_All**	**XP_009356755.1**	**Adenylate isopentenyltransferase 3 (IPT3**)	**0.063416866**
** Contig4362**	**XP_009362178.1**	**Cytokinin riboside 5′-monophosphate phosphoribohydrolase LOG8-like isoform X1**	**2.013697939**
Ethylene			
** Contig5163**	**XP_009379006.1**	**1-Aminocyclopropane-1-carboxylate oxidase homolog 1-like**	**0.366615713**
** Unigene29055_All**	**XP_009379006.1**	**1-Aminocyclopropane-1-carboxylate oxidase homolog 1-like**	**0.303654342**
TCONS_00019363	XP_009369581.1	Ethylene-overproduction protein 1-like	2.66892483
TCONS_00038593	XP_009379022.1	Ethylene receptor 2	0.464874281
TCONS_00038594	XP_009379022.1	Ethylene receptor 2	0.467595351

Twenty-five genes encoding transcription factors belonging to nine families were also identified as *PpTFL1-1* co-expressed genes (Table 2). Among these genes, the abundance of five genes (TCONS00049929:Aux/IAA, TCONS_00027282:bHLH, TCONS_00031878:C2C2, TCONS_00033627:MYB, and TCONS_00053231:MYB) in developmental stage 0.2 decreased to less than one-fifth compared with stage 0, while no genes with expression increases over five times were found during floral induction (Table 2). Considering that the expression of *PpTFL1-1* in developmental stage 0.2 reduced to 1/23 compare to stage 0, it was assumed that these five genes were potentially involved in *PpTFL1*-mediated floral induction.

**Table 2.** *Transcription factors showed coexpression pattern with* PpTFL1 Values of fold change less than 1 signify up-regulation and values more than 1 signify down-regulation during floral induction. Genes mentioned in the main text are marked in bold. N/A, not available.

Gene id	Annotation in pear genome database in Genbank	TF family (annotated by MapMan)	Fold change (stage 0/stage 0.2)
TCONS_00021856	XP_009356262.1	AP2/ERF	0.4888638
TCONS_00026946	XP_009373164.1	AP2/ERF	0.368891721
Contig793	XP_009364491.1	Aux/IAA	2.026782833
TCONS_00032086	XP_009353580.1	Aux/IAA	2.079878128
**TCONS_00049929**	**XP_009339008.1**	**Aux/IAA**	**11.74525523**
TCONS_00006141	XP_009364248.1	bHLH	2.075441931
**TCONS_00027282**	**N/A**	**bHLH**	**6.762526096**
TCONS_00067982	XP_009373851.1	bHLH	4.036924111
Contig11120	XP_009344647.1	C2C2	2.7925173
Contig1692	XP_009367367.1	C2C2	2.991881548
Contig1693	XP_009359344.1	C2C2	3.330619189
**TCONS_00031878**	**XP_009350854.1**	**C2C2**	**6.558992424**
CL13778.Contig3_All	XP_009379450.1	C2H2	0.413760634
CL12823.Contig1_All	XP_009347054.1	HB-zip	2.752035101
No_Contig170	N/A	HB-zip	1.586132542
TCONS_00000655	XP_009364159.1	HB-zip	0.462477789
TCONS_00013081	XP_009366625.1	HB-zip	2.418063948
TCONS_00027401	XP_009373485.1	HB-zip	0.439047215
Contig1360	XP_009337966.1	MADS	0.389380572
Contig7440	XP_009361730.1	MYB	2.742035043
**TCONS_00033627**	**XP_009362719.1**	**MYB**	**9.30428067**
**TCONS_00053231**	**N/A**	**MYB**	**11.11264478**
TCONS_00026858	XP_009373203.1	WRKY	2.087081148
TCONS_00041795	XP_009334707.1	WRKY	2.845753289
TCONS_00064090	XP_009375805.1	WRKY	0.380635681

### Confirmation of the expression patterns of the selected genes in floral development

**Fig. 8.**
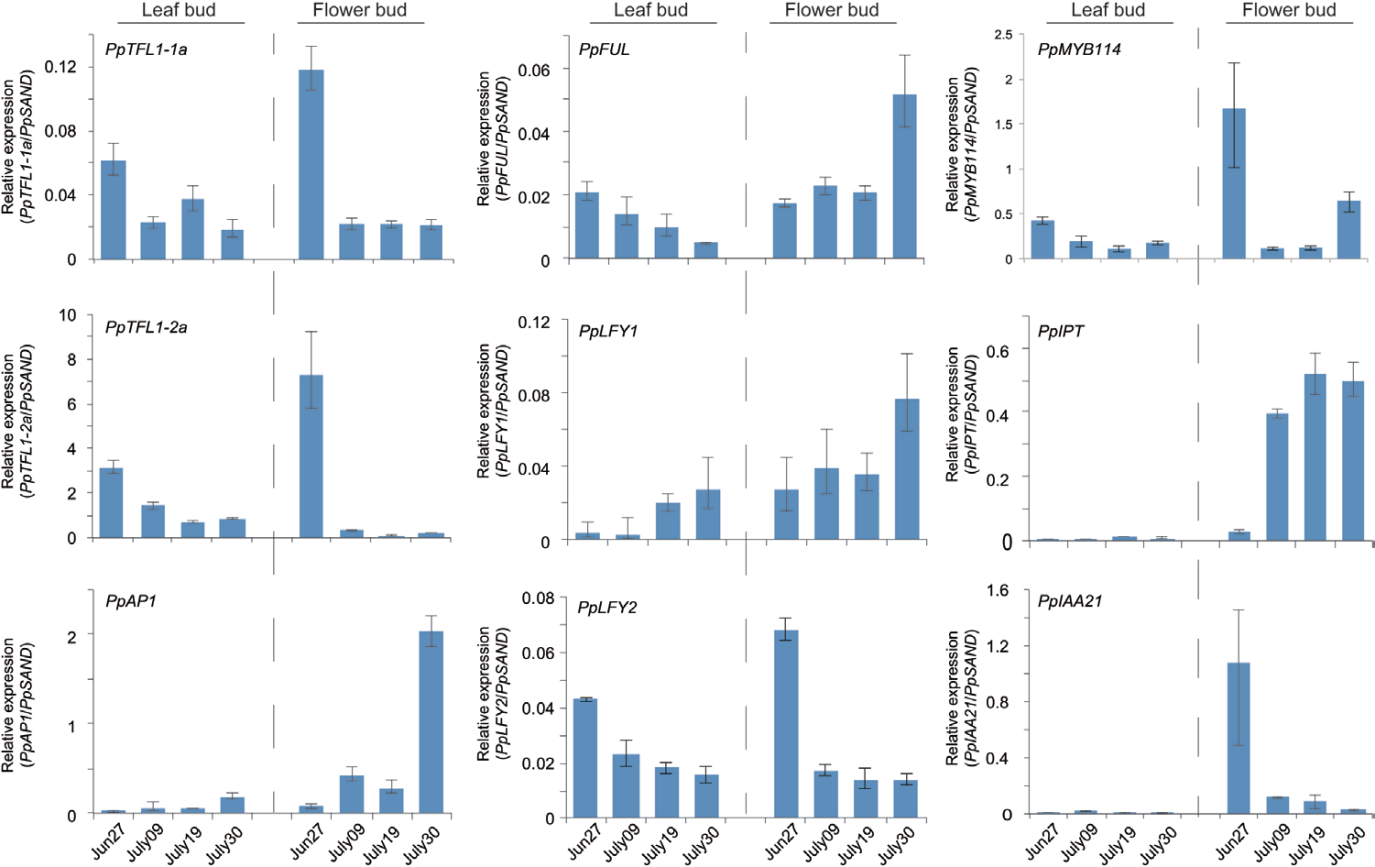
Relative expression levels of selected genes in flower and leaf buds of the newly grown long shoots during floral development in 2013. Data are presented as averages of samples with the same date. Error bars indicate standard error of three technical replicates. (This figure is available in color at *JXB* online.)

As our RNA-seq data came from the transcriptomes of flower buds grown in a natural environment, it is impossible to discriminate whether the differential expression was essentially due to floral development or purely to changes in environmental cues. Therefore, the expression patterns of selected plant hormone-related genes and transcription factors were compared between the buds destined to flower (apical buds in the newly grown long shoots) and leaf buds (lower positioned buds in the newly grown long shoots) (Fig. 8). The abundance of *PpTFL1-1a* and *PpTFL1-2a* transcripts rapidly decreased in flower buds, but this decrease was not obvious in leaf buds. Similarly, high expression levels of genes related to floral differentiation, including *PpAP1* and *PpLFY1*, were observed only in the flower buds, although *PpLFY2* (Esumi *et al.*, 2007) showed the opposite expression pattern. In addition, some other genes, such as *PpMYB114*, *PpIPT*, and *PpIAA21*, also showed different expression patterns between the flower and leaf buds, suggesting these genes are possibly involved in the regulation of floral development.

**Fig. 9.**
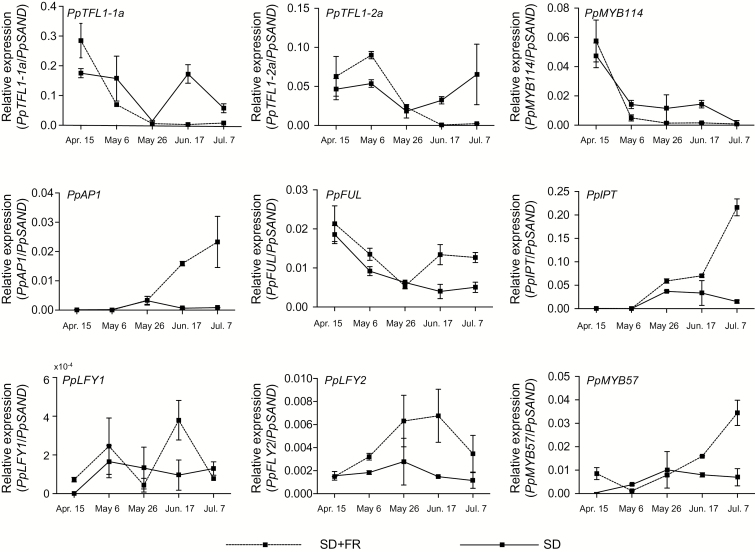
Relative expression levels of selected genes during far-red irradiation. Far-red light treatment induced the flower bud formation as described in Ito *et al.* (2016). SD, short-day treatment; FR, far-red irradiation. Error bars indicate standard error of two biological replicates.

To reconfirm whether these genes were involved in the regulation of floral development, we monitored their expression during far-red light-induced floral induction (Ito *et al.*, 2016). Because of the limited sample amounts, we selected the following genes: *PpTFL1-1a*, *PpTFL1-2a*, *PpAP1*, *PpLFY1*, *PpFUL*, *PpIPT*, *PpMYB114*, and *PpMYB57*. Far-red light accelerated the down-regulation of the *PpTFL1* gene. The expression of *PpMYB114* was also inhibited by far-red light irradiation. Conversely, transcriptions of *PpAP1*, *PpIPT*, and *PpMYB57* increased, suggesting the involvement of these genes in floral development (Fig. 9).

## Discussion

### Down-regulation of *PpTFL1* mediated pear floral induction

Several previous studies have reported an increase in *FT* expression during floral induction in fruit trees such as Satsuma mandarin (Nishikawa *et al.*, 2007), grapevine (Carmona *et al.*, 2007) and mango (Nakagawa *et al.*, 2012). In apple, the expression of *MdFT1* (*PpFT1a* homolog in apple) increased concurrently with floral induction in the apical meristems of fruit-bearing shoots (Kotoda *et al.*, 2010). Hättasch *et al.* (2008) demonstrated that the transcript accumulation of apple *MdFT1* in the vegetative apical bud begins 1–2 weeks earlier than the first visible morphological changes. These results demonstrated the important role of *MdFT1* in the control of flowering time. Considering the close genetic relationship between pear and apple, we expected to see a similar expression pattern for *PpFT1a* in Japanese pear to that of its apple homolog. Contrary to our expectations, *PpFT1a* was not up-regulated during floral induction in the apical tips/buds of the newly grown long shoots that are destined to develop to flower buds (Fig. 1B), although it could rescue the Arabidopsis *FT* mutant *ft10*. Neither was the induction of *PpFT2a* observed in pears (Fig. 1B). Kotoda *et al.* (2010) demonstrated that *MdFT2a* (*PpFT2a* homolog) is mainly expressed in reproductive organs, including flower buds. Therefore, we assumed that *PpFT2a* would also possess a wide range of functions, including regulation of floral development. Floral induction takes place without apparent induction of *FT* mRNA in the meristems of pear. Although the reason for the differences in *FT* expression between pear and apple at the floral induction stage remains unknown, the differences in inflorescence (determinate inflorescence in apple *versus* indeterminate inflorescence in pear) may be related to the difference in behavior of *FT*. In addition, it cannot be excluded that other genes, including possible *FT*-like genes, function as a flower inducer in pear, which needs further study.

Although the expression of *PpFT* did not significantly increase prior to visible floral initiation, the expression of two *PpTFL1* genes declined sharply during floral induction (Fig. 2A). RNAi silencing of pear *TFL1*, *PcTFL1-1*, and *PcTFL1-2* has been shown to be associated with an early flowering phenotype in European pear (Freiman *et al.*, 2012). Esumi *et al.* (2007) showed that pear *PpTFL1* transcripts accumulated in the buds during the vegetative stage, but decreased significantly during floral induction in another Japanese pear cultivar, ‘Hosui’. Likewise, in octoploid strawberry (*Fragaria* × *ananassa*), which also belongs to the family Rosaceae, high expression of *FT* was not recorded in leaves, but *TFL1* expression decreased in the meristem under flower bud induction conditions (short day and low temperature), demonstrating an important role of *TFL1* in flowering in octoploid strawberries (Nakajima *et al.*, 2014). Moreover, because dysfunction of *TFL1* is correlated with the continuous flowering phenotype in modern rose and woodland strawberry (*Fragaria vesca*), *KSN* (*TFL1* homolog) has been implicated in maintaining vegetative growth and modifying flowering seasonality (Iwata *et al.*, 2012). The phylogenetic tree analysis showed that the PpTFL1s studied in the present work are the homologs of FvKSN but not of other TFL-like proteins. Thus, we suggest a similar role for *PpTFL1* in pear floral induction through a transcriptional reduction (Fig. 2A) as observed in strawberry and rose.

The role of TFL1 in controlling the floral induction is mediated by its activity as a transcriptional corepressor through the interaction with the bZIP-type transcription factor FD (Abe *et al.*, 2005). As TFL1 and FT have been suggested to compete for the corepressor FD and regulate flowering time (Ahn *et al.*, 2006), we speculated that the resultant reduction in *PpTFL1* might change the balance of competitive binding with FD, which would lead to a relative increase of the FT–FD complex. The *FT*/*TFL1* ratio was also proposed to be important for the control of flowering time as well as for maintaining plant architecture in tomato and maize (Shalit *et al.*, 2009; Danilevskaya *et al.*, 2010). Recently, it was reported that apple *MdTFL1-2* plays a key role in alternate bearing and return flowering, which is in accordance with our conclusion (Haberman *et al.*, 2016). As *PpTFL1* is spatially and temporally associated with pear floral induction, we attempted to investigate how *PpTFL1* was regulated during *PpTFL1*-mediated floral induction using RNA-seq.

### Possible regulation of *PpTFL1* in floral induction

Floral induction is regulated by both endogenous and exogenous cues, including photoperiod, vernalization, autonomous GA, thermosensation, and aging pathways. In a recent transcriptome analysis, sugar and plant hormone-related genes, including CK, ABA, and GA, were shown to be involved in flower induction in apple (Xing *et al.*, 2015). We found that the expression patterns of *ABA8h′*, *LOG*, and *YUCCA* were correlated with that of *PpTFL1*. In addition, biosynthesis and signaling genes related to auxin, ABA, and ethylene were also identified as *PpTFL1* co-expressive genes. Generally, the expression of auxin and ABA metabolism and signaling genes tended to decrease during floral induction, while gene expression in ethylene pathways tended to be activated (Table 1). In model plants, *TFL1* directly functions in the repression of flowering-related MADS-box genes, such as *AP1*, *FUL*, and *LFY* (Bradley *et al.*, 1997; Ratcliffe *et al.*, 1999; Hanano and Goto, 2011). Our results also showed that the expressions of *PpAP1*, *PpFUL*, and *PpLFY1* were up-regulated after the down-regulation of *PpTFL1*, indicating that they probably function downstream of *PpTFL1*. Conversely, the expression of plant hormone-related genes concomitantly changed in a synergistic or antagonistic way to *PpTFL1*, which may suggest that they function upstream of *PpTFL1* or in a *TFL1*-independent manner.

In Arabidopsis, GA positively regulates flowering mostly through direct up-regulation of the floral integrators *LFY*, *SOC1*, and *AGAMOUS*-*LIKE 24* (*AGL24*) in an FT-independent pathway (Blázquez and Weigel, 2000; Moon *et al.*, 2003; Liu *et al.*, 2008). Additionally, ABA inhibits flowering in a DELLA-dependent pathway, during which no *FT* or *SOC1* induction has been observed (Achard *et al.*, 2006). Furthermore, ethylene also delays flowering through interactions with the GA pathway by reducing endogenous GA levels (Achard *et al.*, 2007). However, we did not observe coexpression of GA-related genes with *TFL1* or activation of *PpLFYs* at stage 0–0.2, suggesting that these hormone-related DEGs may not be involved in GA-induced flowering in pear. By contrast, much fewer studies have been reported on the regulation of *TFL1*; it is still not well characterized as to how internal and/or external cues affect *TFL1*, even in model plants. In pear, expression of *PpFT* was not induced prior to visible floral initiation; additionally, the expression of *PpLFY1*, *PpSOC1*, and other genes related to floral development also lagged from floral induction. By contrast, the expression of *PpTFL1* seems to be more important than *PpFT* for floral induction. It is possible that internal and/or external cues transcriptionally regulate *PpTFL1*, which further influences floral induction, suggesting the involvement of these plant hormone-related genes in floral induction.

As a low transcription level of *PpTFL1* is potentially required for transition to flower buds, the regulatory mechanism by which *PpTFL1* is transcriptionally repressed needs further discussion. In Arabidopsis, the main floral meristem identity genes, *AP1* and *LFY*, act as repressors of *TFL1* via direct binding to the promoter and function during flower development (Kaufmann *et al.*, 2010; Moyroud *et al.*, 2011; Winter *et al.*, 2011). In addition, MADS-box transcription factors *SOC1*, *SHORT VEGETATIVE PHASE* (*SVP*), *AGL24*, and *SEPALLATA4* (*SEP4*) are also proposed to function as repressors of *TFL1* in developing flowers and are essential for repressing the activity of *AP1* and *LFY* (Liu *et al.*, 2013). However, these MADS-box genes were only differentially expressed after floral initiation in our study (Figs 2, 5, 8 and 9). Our results demonstrated that *PpTFL1* expression decreased rapidly prior to visible floral initiation, while *PpAP1* expression remained at a low level and started to increase after floral initiation showing high expression at later stages of floral differentiation. Considering that *PpSOC1* was constantly expressed during floral development, we inferred that the repressive effects (if any) of these genes on *PpTFL1* expression would take effect not during the floral induction stage but during the floral initiation/differentiation stages in pear. Further studies on the regulatory pathway for the repression of *PpTFL1* prior to visible floral initiation will be able to provide more interesting insights.

In Arabidopsis, *cis*-elements for *TFL1* are located in both the 5′ intergenic region and 3′ intergenic region in a functional modular structure (Ohshima *et al.*, 1997; Kaufmann *et al.*, 2010). Accordingly, the 5ʹ proximal region mainly contains elements controlling the expression level, while the 3ʹ intergenic region contains several modules responsible for the spatiotemporal expression of *AtTFL1* (Serrano-Mislata *et al.*, 2016). As transcription factors act as repressors or enhancers depending on the context, it is reasonable to propose that other transcription factors also play crucial roles in the down-regulation of *PpTFL1*. In our datasets, several transcription factors such as MADS-box, WRKY, and HD-Zip factors showed expression patterns consistent with that of *PpTFL1* (Table 2), suggesting their possible regulatory functions in floral induction. It is possible that these transcription factors function as intermediaries to mediate *PpTFL1* repression induced by light, hormones, and other cues.

## Conclusion

Similar to the observation in strawberry and rose, the down-regulation of pear *PpTFL1* is associated with floral induction, suggesting a conserved mechanism, with the reduction of *PpTFL1* expression as the main cause of this step. Plant hormone-related genes and several transcription factors belonging to different families may be involved in the repression of *PpTFL1*. Thus, the current results provide a new platform for more in-depth investigation of the gene repertoires participating in the regulation of *PpTFL1* expression. In addition, because of the complex localization and structure of *PpTFL1*, it is necessary to determine the exact *cis*-elements involved in *PpTFL1* regulation, which will be the basis of further investigation on the direct *trans*-factors of *PpTFL1*. However, we still could not exclude the possibility that *PpFTs* and *PpTFL1* participate in the regulation of floral induction at the post-transcriptional or (post-) translational level. Further investigations on this topic are also needed.

## Supplementary Data

Supplementary data are available at *JXB* online.

Fig. S1. Photographs of the newly grown long shoots and spur of ‘Kosui’.

Fig. S2. Morphological change of apical meristem during the floral development in ‘Kosui’.

Fig. S3. Phylogenetic tree of PpFTs, PpTFL1s, and PpCEN-like with their homologs of other species.

Fig. S4. *PpFT1a* can rescue the late flowering phenotype of the *ft10* Arabidopsis mutant derived from *col0*.

Fig. S5. Relative expression levels of *PpFT1a*, *PpFT2a*, *PpTFL1-1a*, *PpTFL1-2a*, and *PpAP1* in the apical buds of the spur during floral development in ‘Kosui’ in 2009.

Fig. S6. Relative expression levels of *PpFT1a* in the leaves and stems of the spur during floral development in ‘Kosui’ in 2009.

Fig. S7. Functional characterization of *PpTFL1-2a* using the Arabidopsis mutant *tfl1-13*.

Table S1. Primers used in this work.

Table S2. Summary of RNA-seq data.

Table S3. qRT-PCR confirmation of the reliability of RNA-seq results.

## Funding

This work was partly supported by the Japan Society for the Promotion of Science (JSPS; grant no. 15K14659 to T.M.) and National Natural Science Foundation of China (grant No. 31501736 to S.B.).

## Supplementary Material

Supplementary_Tables_S1

Supplementary_Tables_S2

Supplementary_Tables_S3

Supplementary_Figs_S1_S7

## Abbreviations:

ABA: abscisic acid
ACO: 1-aminocyclopropane-1-carboxylate oxidase
AP1: APETALA1
CAL: CAULIFLOWER
CK: cytokinin
DEG: differentially expressed gene
FT: FLOWERING LOCUS T
FUL: FRUITFUL
IPT: adenylate isopentenyltransferase
LOG: cytokinin riboside 5′-monophosphate phosphoribohydrolase
NCBI: National Center for Biotechnology Information
NCED: 9-*cis*-epoxycarotenoid dioxygenase
RPKM: reads per kilobase per million mapped reads
SOC1: SUPPRESSOR OF OVEREXPRESSION OF CONSTANS 1
TFL1: TERMINAL FLOWER1
WGCNA: weighted gene coexpression network analysis
YUCCA: indole-3-pyruvate monooxygenase.
