# Supplementary material for: Repression of TERMINAL FLOWER1 primarily mediates floral induction in pear (Pyrus pyrifolia Nakai) concomitant with change in gene expression of plant hormone-related genes and transcription factors
Source: J Exp Bot. 2017 Sep 7;68(17):4899–914. doi: 10.1093/jxb/erx296 (PMC5853822; doi:10.1093/jxb/erx296)
Supplement: Supplementary_Figs_S1_S7 [file erx296_suppl_supplementary_figs_s1_s7.pdf]

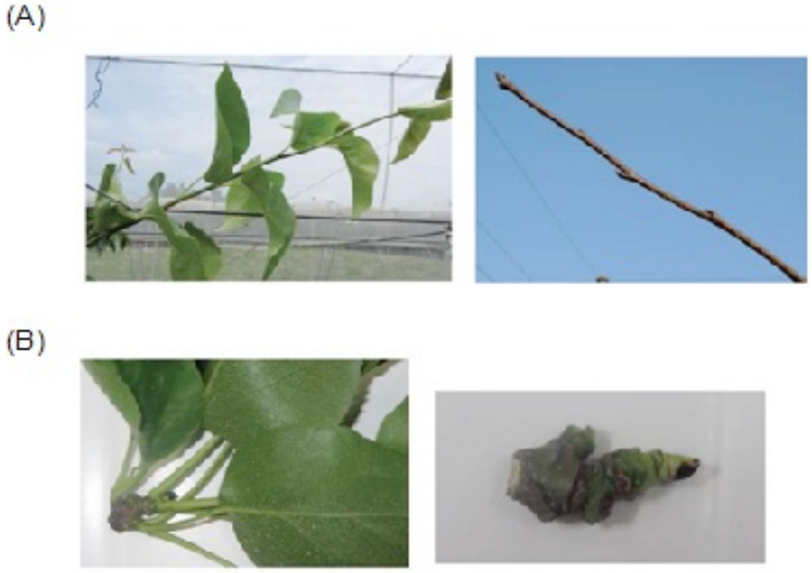

Supplementary Figure S1.
Photographs of the newly grown long shoots and spur of Japanese pear ‘Kosui’. (**A**). Photographs of the newly grown long shoots in summer (left) and winter (right). Apical buds develop into flower buds and several lateral buds near the top can form the flower buds, while others develop to leaf buds. The number of the flower buds varies among shoots largely based on their nutritional conditions. (**B**). Photographs of the spur with (left) or without (right) leaves in summer. Apical buds develop into flower buds. The buds were used in Supplementary Fig. S5 and the leaves and stems (very short; epiphytic portion of leaves) were used in Supplementary Fig. S6.

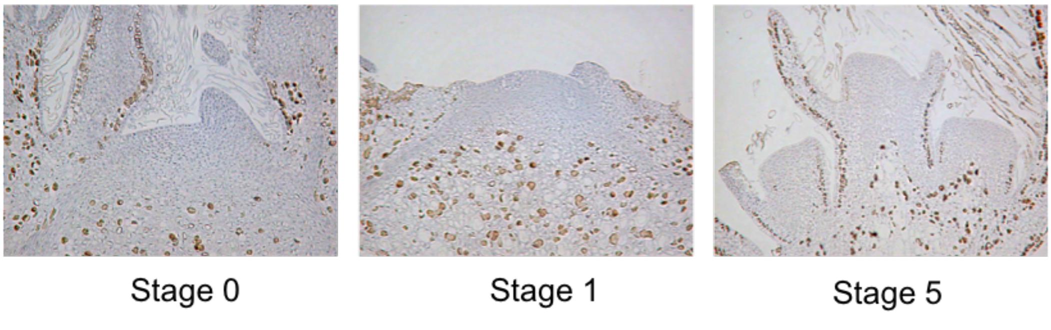

Supplementary Figure S2.
Morphological change of apical meristem during the floral development in the Japanese pear ‘Kosui’.

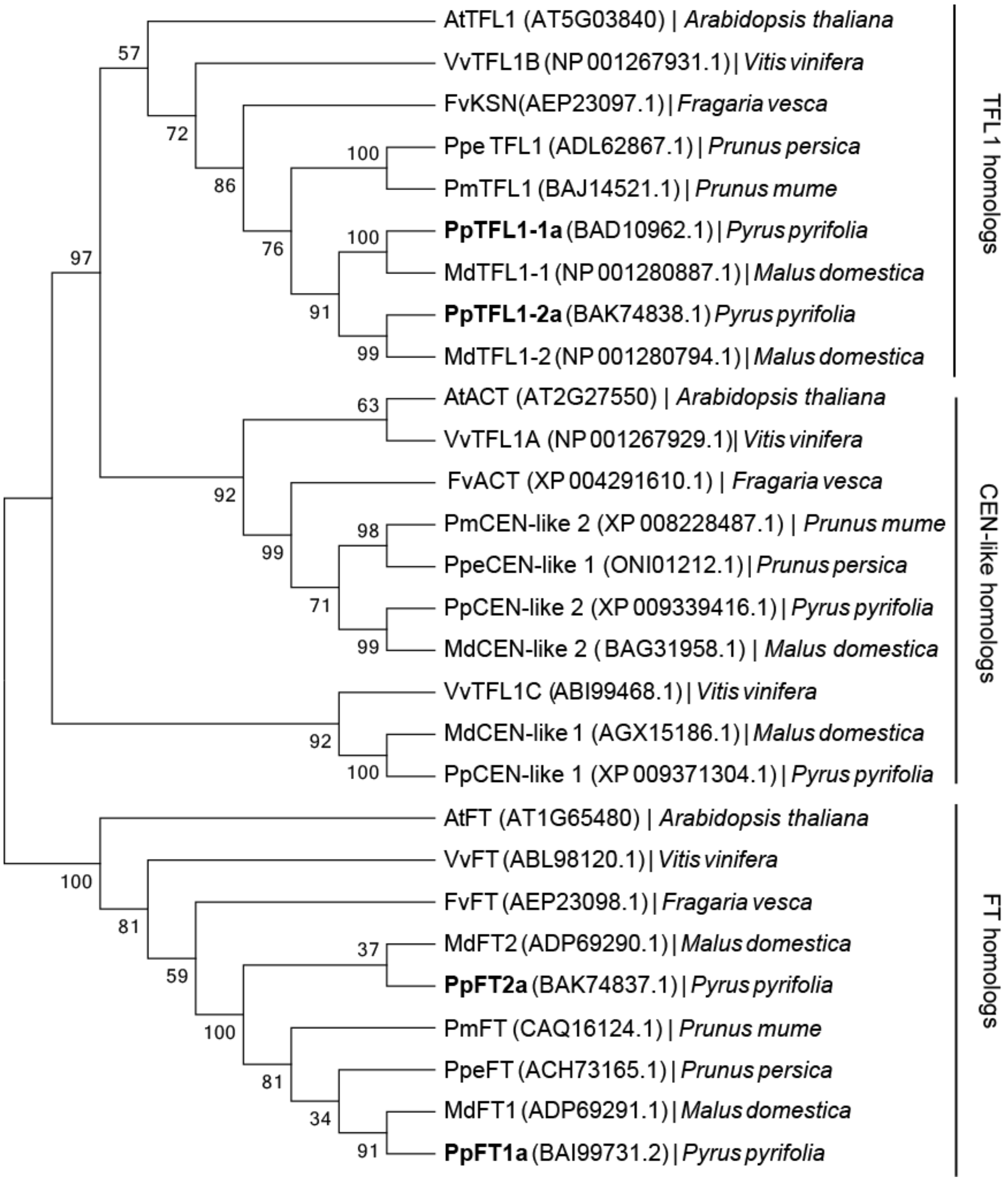

Supplementary Figure S3.
Phylogenetic tree of PpFTs (PpFT1a and PpFT2a), PpTFL1s (PpTFL1-1a and PpTFL1-2a), and PpCEN-like with their homologs of other species. The genes studied in the present work were marked as bold. The phylogenetic tree was produced by MEGA7 using the neighbor-joining method with the bootstrap test of 1,000 replicates.

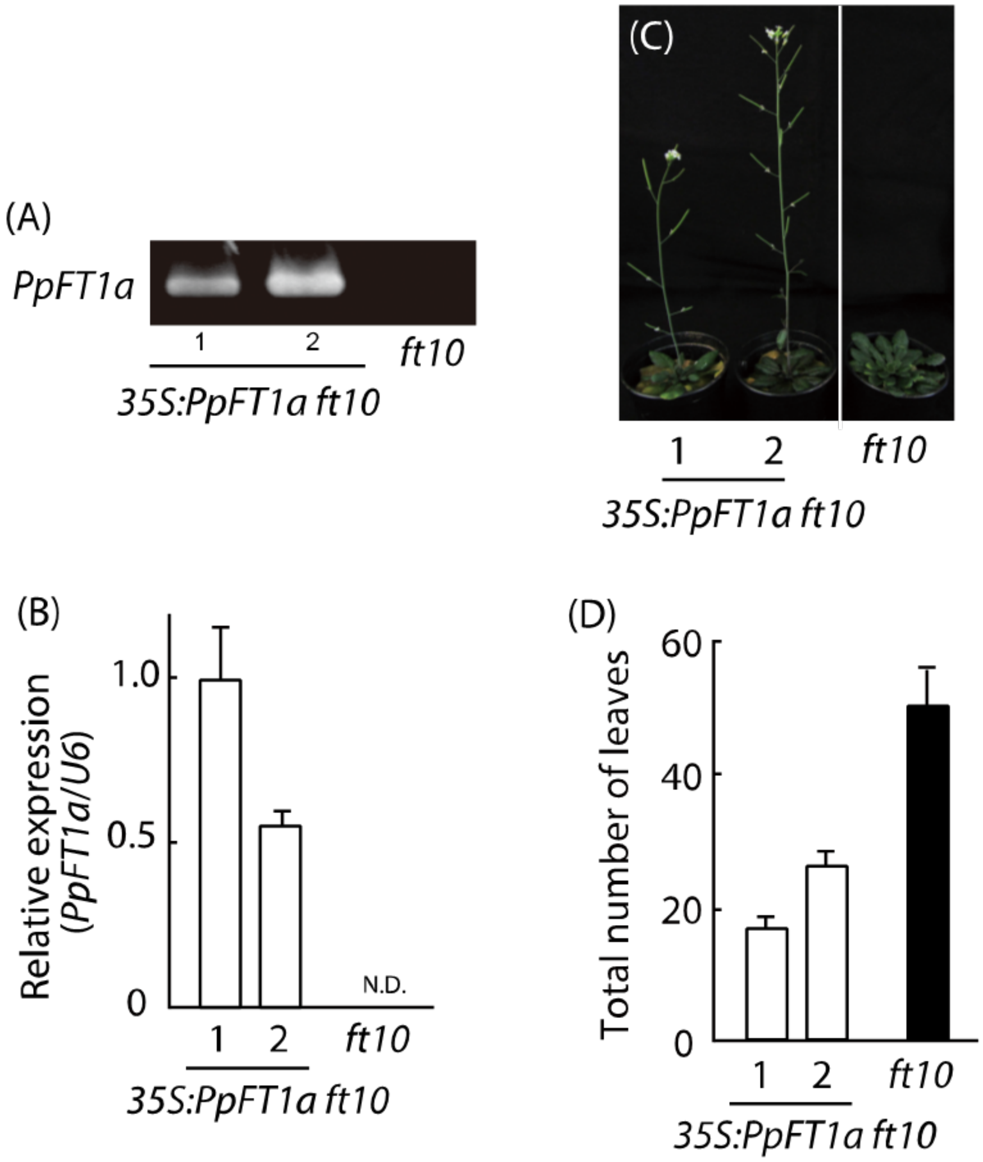

Supplementary Figure S4.
PpFT1a can rescue the late flowering phenotype of the *ft10* Arabidopsis mutant derived from *col0*. PCR identification of two independent lines of *35S:PpFT1a/ft10* (A). Expression level of *PpFT1a* in the two complementation lines (B). Phenotypes of two complementation lines along with *ft10* (C), and the flowering times of the two complementation lines (D); both two complementation lines flowered after 50 days under a 16h/8h photoperiod, but *ft10* did not. Error bars showed the standard error of at least five biological replicates.

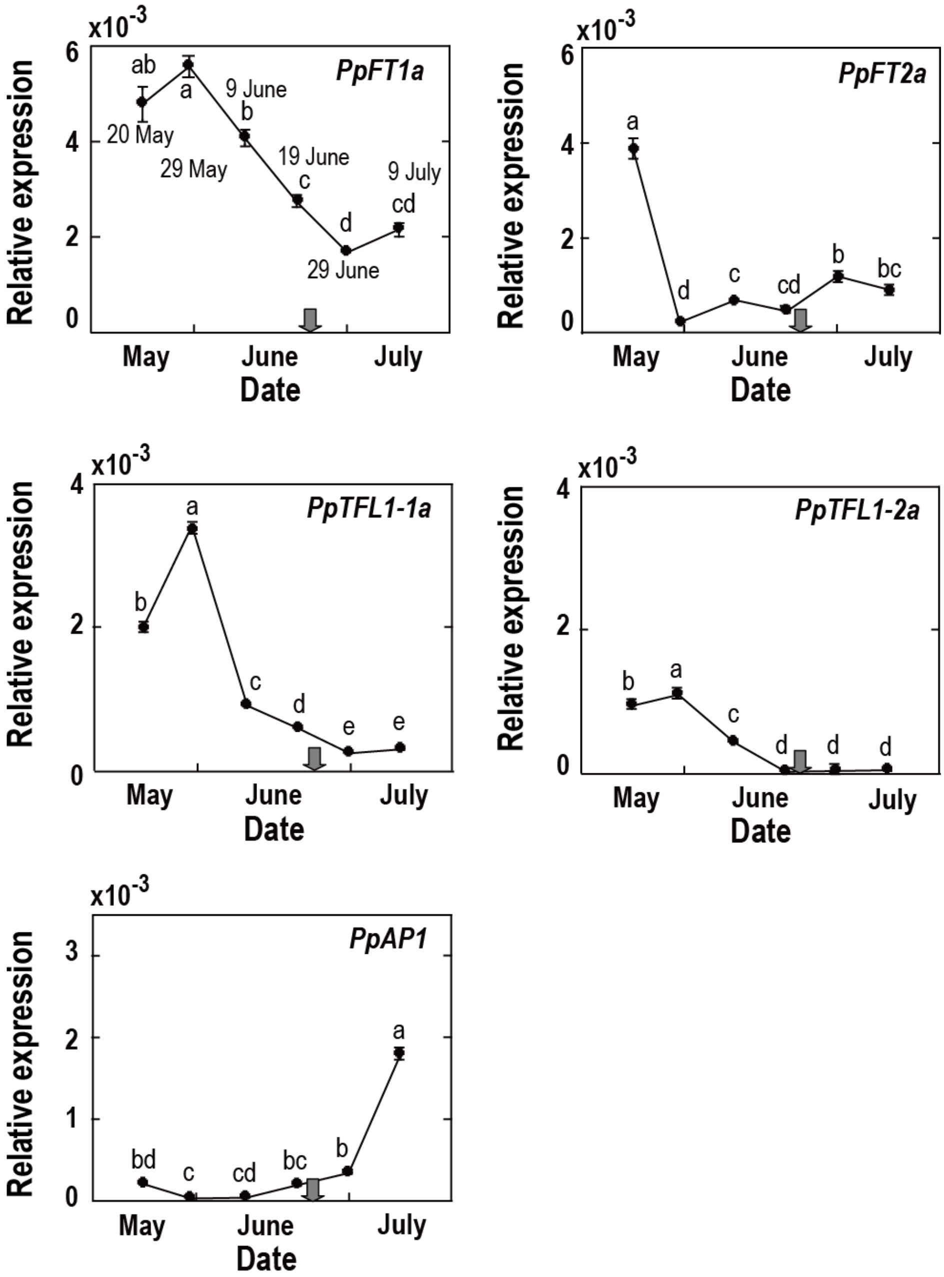

Supplementary Figure S5.

Relative expression levels of *PpFT1a*, *PpFT2a*, *PpTFL1-1a*, *PpTFL1-2a*, and *PpAP1* in the apical buds of the spur during floral development in 'Kosui' in 2009. Error bars represent the standard error (n=3). Same letters are not significantly different at $P < 0.05$. Arrow indicates stage 1.

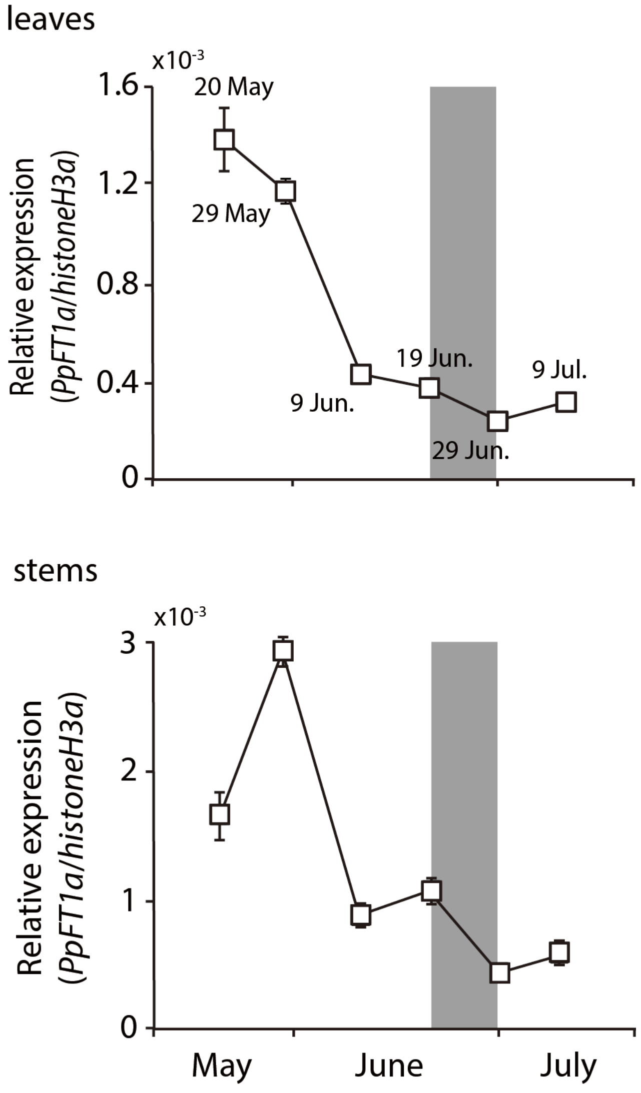

Supplementary Figure S6.

Relative expression levels of *PpFT1a* in the leaves (upper) and stems (lower) of the spur during floral development in ‘Kosui’ in 2009. The stage that dome-like structure (= occurrence of visible floral initiation) appeared is indicated by the grey zone. Error bars represent the standard error (n=3).

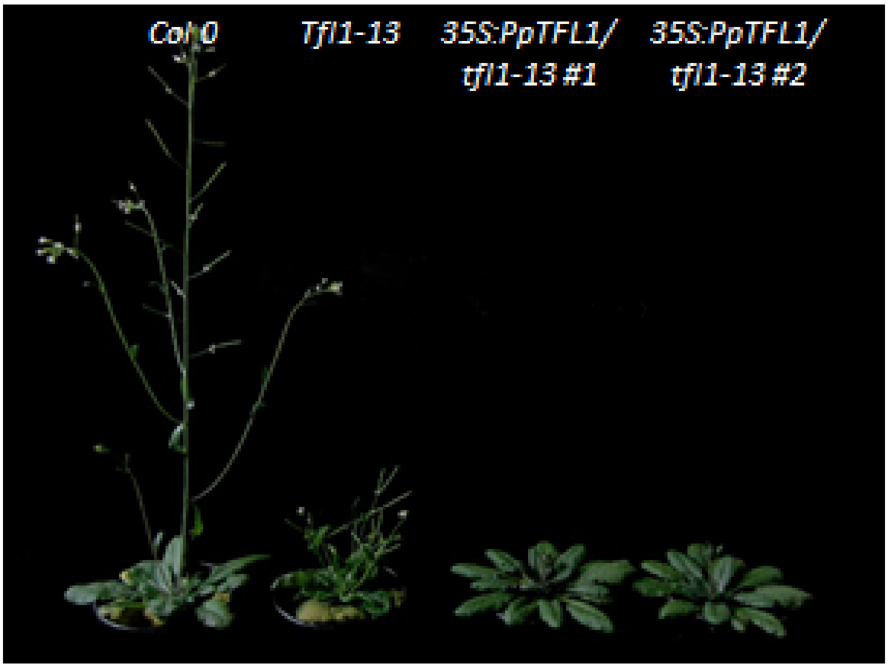

Supplementary Figure S7.

Functional characterization of *PpTFL1-2a* using the Arabidopsis mutant, *tfl1-13*. Early flowering phenotype of *tfl1-13* was canceled in the *35S:PpTFL1/tfl1-13 #1* and *35S:PpTFL1/tfl1-13 #2*.
